# Address to the Graduatiug Class of the Medical Department of Niagara University

**Published:** 1888-05

**Authors:** Henry Flood

**Affiliations:** Elmira, N. Y.


THE BUFFALO Medical AND Surgical Journal

Vol. XXVII. MAY, 1888. No. 10

### Original Addresses.

### *ADDRESS TO THE GRADUATING CLASS OF THE MEDICAL DEPARTMENT OF NIAGARA UNIVERSITY.*

By HENRY FLOOD, M. D., of Elmira, N. Y.

*Most Worthy Chancellor, Ladies and Gentlemen:*

The diploma which has been conferred by this University upon you, gentlemen of the graduating class, gives you a legal right to commence the practice of medicine. It certifies to the public that you have pursued such prescribed courses of study, and have passed successfully such rigorous tests, that you are proved to be worthy to be members of the brotherhood of the profession of medicine.

The laws of this college make the work of preparation for the honors of this day so long and so thorough, that the degree of M. D. is not to you an unmeaning title.

When you received the degree of "Doctor of Medicine," you surrendered, to some extent, your student's life, and commenced a new and very different one.

Judging from the exhaustive examination which you have passed before the Board of Examiners to-day, I know that you are far better prepared than most beginners, and I assure you that the better the foundation you have laid as a student, the more rapid and successful you will be in rearing the superstructure of a physician's life.

When I say you surrender to a certain degree your student's life, I mean to a limited degree only; for, if you desire to compete successfully with other practitioners, and keep abreast of the progress of medical sciences, you must always be students, and hard students at that.

The first years of your professional life, before you have established a reputation and acquired a large practice, will afford you golden opportunities to broaden and perfect your medical education.

In after years, when you have acquired a large practice, the urgent duties of your profession will make study both irregular and difficult. Neither interruptions nor overwork can be avoided, and when you come home wearied, it will not be easy to turn to your books, and concentrate your distracted thoughts on some medical subject; nor will you then feel inclined to read many pages of chaff in order to sift out a few pages of wheat.

You must not assume, because you have your degree and have been good students, that you know it all. I will tell you now, you will never know it all.

I adjure you not to commence life with the conceit of some graduates—that they know just a little more than anybody else—for if you do, the day is not far distant when you will find, to your mortification and chagrin, that some old and seedy-looking doctor will be able to see you and go you one or two better.

You should remember, too, that you have been studying only the theoretical part of medicine. You may have your rules learned, word for word; you may be able to name every bone, muscle and ligament in the body, to give the symptoms of every disease; but, when you come to apply your book knowledge to certain cases, you will often find yourself sadly confused.

You must now begin to apply yourself to studying cases. The practitioner who has the most experience, and knows how to utilize it, is bound to be successful.

The most successful, however, are those who always remain students, adding every year to their stores of professional knowledge, and utilizing these fresh acquisitions according to the dictates of judgment and the guidings of experience.

There are some practitioners who, as soon as they glance at their patient, see instantly whether or not he is better, and are very seldom in the wrong in this first impression. Their practical knowledge, experience and skill have taught them what no books can teach.

Some men thus know, intuitively, what others can gain only by hard labor. They are naturally adapted to the practice of medicine, and their work is easy, gratifying and successful.

When you begin your work, take time to investigate your cases, to analyze their symptoms, to devise a methodical way of attending to your business, and to hold yourself to a thoroughness that will give you close insight into your practice. Such a beginning will give you a splendid experience, the great teacher in all professional life.

The habits a physician acquires in the first years that follow his graduation, will stay with him through life, and determine his professional character.

If you are careless and slip-shod in the beginning, you will generally remain so. If you are careful and methodical, these habits will assist you later on, when time becomes very valuable, and when your wits are taxed to so economize it that you can attend to the pressing demands of a growing practice.

To the busy physician, economy of time is one of the great problems that is always staring him in the face.

If you desire to be successful, you must attend to your business faithfully and diligently, and never neglect a case. Negligence, in the eyes of the law, is a criminal offense.

Many a physician who has been sued for malpractice has lost the accumulation of a life-time by having neglected one patient. The law holds that the general practitioners need show only ordinary skill and knowledge in the treatment of a case; but nothing will excuse even the most skillful, expert and educated physician, if it is proven that he has been guilty of negligence.

Let me now call your attention to what seems to me one of the most important matters for your consideration—that is, your charges.

The services you render society, ministers to its good, and deserves compensation. The service is often laborious, difficult, unpleasant, and always requires years of previous study; such service is not mere manual labor, and it may reasonably ask for high compensation.

Most physicians, sooner or later, have families that are dependent on them for support; and how many widows and orphans have been left by physicians nearly poverty-stricken, simply because he who ought to have been their provider, was a careless collector and a poor business man, and, for the sake of others, really sacrificed his wife and children. Every physician should so provide for his family, that, in the event of his death, he should leave means for their support in a manner suitable to their station in society.

Many physicians become so absorbed in their profession, that they neglect their accounts, lose many bills which could be collected, and let their finances run so loose, that when they die, their accounts are so mixed that they are nearly worthless.

From a logical standpoint, there is no reason why a physician should not receive pay for what he does.

The ordinary practitioner is not a missionary—not a member of a charitable fraternity. He is a civilian, and it is his legal and moral duty to support himself and those dependent on him. He has no more right to give away his time than a grocer his goods, or a baker his bread.

Some say it is severe for a physician to refuse to attend a suffering man because he has no money. The grocer and the baker are not asked to feed the starving. They send them to the city and county authorities for necessary food, fuel and clothing, and they say that they contribute their share towards the support of the poor in the taxes which they pay to the community. So does the physician, as a good physician, pay his share, and, as a good husband and father, he should refuse the imposition of paying more.

I would not advise any physician to go as far as the grocer or the baker, nor would he be justified in so doing by public sentiment; for, a profession should rise above the mere basis of making money.

A profession is something more than a mere business. It has honorable obligations of public service far higher than the precept of the store and the shop.

The deserving poor very seldom suffer for the care of a physician, for all of us are charitable enough to give them our time, money and services, and I doubt that you, any more than I, will let any person in actual need suffer.

There is, however, a class that will live better than you, indulge in luxuries that you will not even think of, dress better than you, who will never pay you their bills. They are not charity patients. They are dead-beats, and they beat no one more than the physician.

They are unreasonable, exacting; claim your constant attendance; call for you unnecessarily at night. Why? For it matters little to them whether or not they have a large bill, for they have no idea that they will ever pay it, and when you ask them for your dues, they turn upon you with criticism and abuse, and trump up some excuse for the avoidance of your just claim.

They live beyond their means. They use fraudulently the money that belongs to others. What you have earned by hard labor, they dishonestly withhold, and spend for their own gratification.

When you have a bill against a man, and cannot collect it, and you see this man or his family wearing better clothing than you, or if you see him attending the theater, there is something wrong about him, for if he can afford to wear fine clothing, and dress his family extravagantly, he can afford to pay you. If he has money enough to buy theater tickets, he should pay his debts. If he can smoke good cigars, and spend money treating his friends, he should pay his doctor's bill. If he calls on you, it is unmistakably your duty to refuse to attend him until he settles his accounts.

This class—not a small factor of our community—will not pay you if it can be avoided, but if they find that they must pay, or not receive your services, many will be honest by necessity, while others will hasten to the doctors who will stand beating.

I can recall a physician who recently died, that was very economical; neither drank, smoked, nor spent money in any manner for luxuries. He had practiced forty years, and had a very extensive business. When he died, he left only five or six thousand dollars to his wife and family.

This man gave away the larger portion of his life's work. His family were left with a scant support, while some of his creditors lived in luxury. I do not hesitate to assert, that if I should try to raise a hundred dollars for a monument for this worthy man's grave from those that were owing him, I would find there would not be enough gratitude in their selfish hearts to contribute towards it a paltry dollar.

It is no charity to give to such persons your services. Your time and strength are too precious to waste on such dishonest and ungrateful wretches. When you do it knowingly, you encourage dishonesty, and are doing yourself, your family and your profession a dire injustice.

I advise you to collect your bills often, and to have a settled system to your finances. When you are unable to collect from certain persons, find out the reason.

If they are honest, industrious and frugal people, and from some ill-luck are unable to pay, balance the books, and charge it up to charity. But if these creditors are idle, extravagant and dishonest, and will not pay you, follow them up relentlessly, with every means that the law provides for your protection.

You are beginning a life in which you will be able to benefit society in ways almost innumerable. If conscientious and patient, you can make your character and influence felt in the house of the rich and poor alike. A good physician, who is a good man, will soon become an influential citizen, and a powerful factor of the community in which he resides, and will win from the public a large measure of respect for his profession.

On the other hand, a physician who is a bad man, is a dangerous man in the community, and can do an immense amount of evil. Such a person not only drags himself down, but causes a general loss of respect for the profession at large, and subjects the honorable practitioner to annoying and undeserved suspicions.

Physicians should be brotherly in their treatment of each other, and avoid in every way unjust criticism, and even when they believe that another physician has erred, they should remember their own fallibility, and avoid, by a judicious silence, wounding the brother's fair name.

It is not honorable to assail anyone's professional reputation by rehearsing to your patients or the public his faults or failures.

The old saying that "doctors always disagree," is too often true. Why? Because they necessarily differ about small details, and some talk so much about these petty affairs, and exaggerate these small differences, that they make mountains out of mole-hills.

Whenever physicians publicly quarrel with one another, the spectacle may be very amusing to people who enjoy witnessing rows—neighbors may urge them on—but you may rest assured that the principals in these professional duels will gain nothing in the esteem of intelligent men and women; but that each will suffer in professional dignity and influence, while often the contest ends in making all concerned the laughing stock of the community.

So it is when the members of the regular practice of medicine take it upon themselves to belittle the practitioners of other schools. The law recognizes them, and the people have good reasons for believing that all physicians have equal rights, and consider your criticisms to emanate from a narrow-minded jealousy. There is no good reason why you should degrade yourselves by following them up with petulant and malicious abuse. Tirades against another school of practice will never strengthen the standing of our own. The common sense of the people estimates these things generally in their true character, and judges the man who indulges in them as a professional scold and a weak mischief-maker.

There are two extremes that practitioners are in danger of rushing into; one lies in the line of an ambition to keep abreast of the times.

This is certainly a laudable ambition, and one of the conditions of any great success; but if not restrained by good judgment and sound discretion, the practitioner will rush wildly from one new remedy to another, never trying any kind of treatment long enough to become thoroughly acquainted with it.

In cautioning you against this temptation, I would advise you, when you find a treatment that is good and reliable, adhere to it as you would to a well-tried friend. Don't desert it for a new one, unless you, by several trials, have proven it to be the better of the two.

On the other hand, there is a greater danger: that is, of getting into a rut, or riding a hobby.

A man who gets into a rut, or rides a hobby, cannot be a very successful man, for his energetic neighbor will run away from him, while he is dreaming over the same old dream.

I knew a professor in a college who had only two remedies: Iodide of potash and quinine, and gave them alike for all diseases. Another, who very seldom gave any other remedy than a hypodermic injection of morphine.

I once had an old physician visit me, who was a finely-educated and accomplished gentleman. He accompanied me on many of my visits. Nearly every time we left a house of a patient, he would try to convince me of the great virtues of calomel, and he thought I was a very poor practitioner because I did not give it to every patient I had.

I soon learned from him what I did not know before—that calomel has an excellent effect in a certain class of cases, but I also learned what, with all his education, he seemed not to know, that in other cases it does more injury than it does good.

Finally, gentlemen, I will end my sermon by advising you to be true to yourselves, to the dignity of your profession, and the honor of your manhood.

Honesty will assist you more than anything else in obtaining your patients' confidence and respect.

Never deceive them, for when a physician once gets the reputation of magnifying his cases, or of concealing their real seriousness, his words either way will have little weight, and at last the lie will defeat itself.

Always endeavor to do what is right, and practice the golden rule both with your professional brethren and your patrons.

Attend with all diligence to the business that you have, and keep abreast with the progress of the medical sciences. If you do, I have little doubt but that you will succeed in the noble work you are about to commence.

May years of usefulness and smiling fortune await you. May the honorable service you shall render to suffering humanity be rewarded with all the generous compensations of money, fame and gratitude that your labors shall deserve.

